# Acne Vulgaris and Its Treatment
1Read before the Bristol Medico-Chirurgical Society, April 12th, 1905.


**Published:** 1905-06

**Authors:** W. Kenneth Wills

**Affiliations:** Physician to the Dermatological Department, Bristol General Hospital


### ACNE VULGARIS AND ITS TREATMENT.[1]

BY

W. Kenneth Wills, M.A., M.B., B.C. Camb.,

*Physician to the Dermatological Department, Bristol General Hospital.*

There seems to be some little vagueness as to what the term "acne" really implies. Some take it to include the comedo, while others describe acne as "pustular inflammation in and around the sebaceous glands and hair follicles." (Crocker.)

There is a pustular condition without any obvious comedo formation, but by far the majority of cases which come before us show the following sequence:—(1) An oily skin; (2) comedones; (3) pustules; (4) "blind boils" with induration round the lesions, the latter sometimes to a marked degree.

This sequence of events, given in the order of observation, is not complete, for there are cases in which circulatory disorder is a marked feature, and is possibly primary. It is in such cases that Malcolm Morris considers the sebaceous obstruction

[1] Read before the Bristol Medico-Chirurgical Society, April 12th, 1905.

to be secondary to an inflammatory process due, as in rosacea, to the local circulatory disorder.

Though this hyperæmia may be the predisposing cause of the acne formation it is not the acne itself, and I would offer as a definition for the disease in question the following:—"An alteration of the contents and secretion of the sebaceous glands, leading to a viscidity thereof with the formation of comedones, and with or without an attendant folliculitis or perifolliculitis."

The microscopical examination of a comedo shows it to be essentially a "hyperkeratosis of the outer third of the pilo-sebaceous follicle." (Macleod.) It is stated that the body itself is formed of epithelial cells, a large amount of grease, and millions of short bacilli, described by Unna, Sabouraud and Gilchrist, and termed by the latter the B. Acnes. (N. Walker.) It is also stated that the bacillus is responsible for the inflammatory condition often attending the comedo, which leads to the formation of pus and also granulomatous tissue. Presumably, as is the case with the inflammatory conditions attending a seborrhœa capitis, the oily secretion acts as a good culture medium for various germs, and the B. Acnes in particular. The latter appears to act by producing sufficient exfoliation of the lining wall of the follicle to interfere with the normal flow of the oil. A cork is thus formed which permits of distension of the gland, and eventually to destruction of it, and, if the process extends through the follicular wall, to a perifolliculitis and the formation of dense masses of granulomatous cells.

The development of acne is commonest in adolescence, and is frequently associated, though I have not been able to demonstrate the invariable connection, with seborrhœa capitis. It may occur in persons otherwise in perfect health, and it may be found in anæmic, unhealthy-looking "weeds." Of the two extremes the former shows a more oily condition, while the latter presents a drier and perhaps more indolent type.

The fact that acne produced by artificial causes, such as tar, &c., affects the same regions as acne arising *per se*, seems to show that the glands affected, viz. those of the cheeks, forehead, nose, chin, neck, back, chest, backs of thighs and arms, have some common peculiarity in structure. It will be observed that they are not connected with the large hairs, but with lanugo hairs; acne does not affect the hairy scalp or the whisker area.

Shoemaker alludes to the kind of sebaceous gland that acne invades as being those with wide ducts to which the lanugo hair is only an appendage, and quotes Pifford in the statement that acne is almost exclusively an affection of this class of gland.

As predisposing causes I place dyspepsia and constipation in the foreground, with a want of fresh-air, exercise, and an unsuitable dietary as in turn the causes of these conditions.

Anything which causes the skin to flush has a harmful action upon an acne already established. Too-hot drinks are the commonest offenders, while alcohol, mustard, pepper and spices, pork, pickles, and pastry should all be regarded with suspicion and eliminated from the dietary. Fats too, as helping in the formation of sebaceous secretion, should be lessened, particularly in my belief the fat of pork and bacon.

In the treatment of the disorder, after the removal of the predisposing causes as far as is practicable, the attention should be directed to the local conditions; and the following considerations should guide us:—

(1) The removal of the superfluity of grease.

(2) The destruction of the irritating micro-organisms growing in the fat.

(3) The enucleation of comedones and pustules.

(4) The alteration of the skin and its secretion in such a way as to make a further infection more difficult.

(5) The maintenance of a more perfect hygiene of the skin.

(i.) To remove the superfluity of grease from the skin it is necessary to relieve the hyperæmia as well as to use mechanical means. From its tendency to relieve congestion and inflammatory conditions ichthyol internally, as well as externally, has frequently given good results. It may be taken in pill form or in capsules, but it must always be supplemented with friction externally. Sulphur internally has probably little effect, though it is excreted to some extent by the skin; it is found that calx sulphurata helps in the resolution of the indurated conditions.

Externally friction with an alkaline medium is of the greatest importance; and although it is objectionable to the patients, the advantage obtained in appearance is so great as a rule that they are willing to persevere. The medium used is generally some form of soap, nor does it matter much what soap; though some skins are so irritated by strongly alkaline soap that perseverance with it would produce a deleterious amount of hyperæmia, and an undue amount of soreness. As friction with a flannel or rubber sponge is an important part of the treatment, soap forms a convenient medium for the alkali, otherwise any mildly alkaline material would do which will remove easily any of the grease which is about the skin. Friction is useful in several ways. It tends to empty the sebaceous follicles, to remove comedones, and to cause the skin muscle fibres to recover their tone, which is frequently wanting.

While this is being done on the face, and I do not wish to dwell upon the already well-known treatment, any attending seborrhœa capitis should receive attention.

(ii.) After the soap-friction some mild antiseptic should be used, and it is a common practice to use sulphur in some form or another. But the antiseptic must be mild enough to prevent undue hyperæmia on its own account. Sulphur, or ichthyol, seem to have a specific action in inhibiting the micro-organisms.

(iii.) Where there are many pustules, the friction is better postponed until they have been dealt with. Incision should be practised, to prevent pock-marking. But expression of the comedones and pustules is not the best method to deal with these. When the patient is willing to give time to himself, a great deal can be done with a sharp surgical needle in lifting out the upper parts of the comedones and in pricking the pustules. I have tried expression and needling, and also drawing out the comedones with a large-barrelled suction syringe. The latter method can scarcely be done on oneself, though the result is good when another does it. It draws out the contents of the follicle without undue bruising of the surrounding tissue. Sometimes it fails to "start" the comedo, but freeing the black-head with a needle will usually make the second attempt successful. I have also used it for small pustules with good results.

Scraping the face with a blunt spatula is a useful practice. This performs several of the offices of friction, but it can scarcely be used where pustules are many and the face is sore.

The shelling of the skin with resorcin I have never practised, but it is said to give good results. In Norman Walker's words, "It consists of the application of equal parts of resorcin and Unna's zinc paste, thickly spread, to the skin twice daily for three or four days. At the end of this period some soothing ointment is applied, and in a day or two the skin peels off in large flakes, bringing with it the hyperkeratotic horny layer and a large number of the comedones." "The method," he continues, "involves confinement to the house, and in that respect is disadvantageous; but it does more in a week than probably two months of the milder treatment will accomplish."

This in one way is altering the skin and its secretion, in that it removes bodily the altered layer and its masses of bacteria. There is, however, another agent to hand which has proved of great service in those cases, which one frequently sees, where ordinary treatment proves too slow to combat the disease in the face of the numbers of comedones that suppurate and indurate in spite of all one can do to prevent them.

In the X-rays we have an agent which alters the skin secretions, stimulates the growth of the normal cells, and promotes the absorption of the granulomatous tissue. So that ulcerations heal, comedones get thrown off with the peeling or powdering skin, and the indurated and blind tumours get smaller and disappear without discharging.

After a satisfactory reaction has been obtained, one usually finds the parts treated by the rays clear of comedones and pustules, showing only the scars of previous abscesses, while outside the rayed area the comedones, papules and pustules are as vigorous as ever.

The good result obtained is not necessarily permanent, but, having got rid of the affection for the time by using more perfect hygiene, the skin may be kept freer in the future. By degrees the skin returns to its ordinary, normal condition, and it rests with the patient himself to keep it clear of infection.

One aims at obtaining a slight erythema, perhaps a little more pronounced than a sunburn. When this is obtained the developing pustules appear swollen and larger, and perhaps a few more make their appearance. A day or two later, however, the skin is seen to be dry instead of oily; the pustules have changed to papules with a scale, which is loosening on its apex; while the comedones are frequently so easily shed that they may be present one day and all gone the next. After this effect has been obtained treatment is still continued for a while, maintaining the erythema, for in so doing a more complete exfoliation of the skin is obtained, and those follicles which have not already taken part in the general process are induced to do so. Perhaps the most noteworthy points are the healing of the gaping craters left by previous small abscesses, and also the lessening and finally the disappearance of the indurations, which are so disfiguring and hard to relieve.

After the process is complete the patient will find that the skin is abnormally sensitive to touch and changes of temperature, in that stroking or entering a hot room will easily produce a blush. But in course of time this dies away, and the skin left is soft, supple, fine, without the gaping mouths of glands, dry, and the scars left by the former pustules have levelled up or their edges have levelled down.

One case I should like to report is that of a man whose face was so badly attacked by acne pustulata et indurata that he could only obtain menial work to do at a low wage. After treatment his face only presented the white patches of old scars, some as big as a finger-nail and numerous smaller ones, and these were smaller than they had been, and no acne spots were to be found. He signalled the success of the treatment by immediately obtaining work to do more fitting his age and ability, and at a large increase of wage. This man was by no means a cleanly fellow, and I saw him several times later, but beyond an occasional spot he showed no attempt to return to his condition. He is now absolutely free of the disease.

Another man was the subject of the worst attack of acne pustulata et indurata on the back that I have ever seen. His work necessitated his carrying sacks upon his back, but he had to give this up owing to the furunculi and deeply burrowing indurated abscesses in quantity. Beyond puncturing a few of the pointing pustules and giving the patient some carbolic acid lotion 1 in 40 to use, I did nothing more than request him to get his back washed with soap and hot water every day, and I treated him with the X-rays. This was one of my early cases, and I took longer over him than I need have done in my caution to prevent an X-ray dermatitis. However, in course of time, he presented a back more like a patient after a bad attack of small-pox, it is true, but clear of comedones, pustules and indurations. I did not continue with the treatment after producing this effect, and he had a recurrence of the condition later, though not nearly as bad. This was relieved in due course.

Perhaps the best case to illustrate the use of the treatment is the following:—

Miss L., sent to me by Dr. Harrison, has suffered from pustular acne for 6 or 7 years. Local and internal medication had only produced a very temporary benefit. She presented a condition of pustular acne over the whole of the face, from the hairy margin to the chin. The skin seemed to run oil, and the chin and the alæ of the nose were very red and full of blind boils.

I started the X-ray treatment Aug. 9th, 1904, carefully, as I always do, to study the patient's tolerance, and it was not till about the tenth application that I obtained anything of a reaction. Then a crop of fresh pustules came out on the chin, and the patient in spite of warning was in despair. The next day (Aug. 26th), however, peeling had started, and was kept going by further doses off and on till Sept. 3rd, when patient left for the Channel Islands. At this time I noted that there were still one or two pustules, the lips were a little sore (I had not protected them), the cheeks and forehead showed marked erythema. She had orders to wash in soap spirit, and bathe with 1 in 20 carbolic lotion when the redness had died away.

I have heard several times from her since, and the result is in every way satisfactory, in that she describes herself (Oct. 28th) as "wonderfully better," and says (March, 1905) the parts treated are remaining well, but there are spots on the parts not treated (in the hair margin). On April 7th she writes that she is as far as she can tell well.

This is a good sample of the ten cases I have treated.

I should not like to close this without alluding to the work of A. E. Wright (of St. Mary's Hospital), probably known to all present, who has treated successfully pustular acne by therapeutic inoculations of staphylococcus vaccine. In this way "he attempts" (to use his own words) "to induce the chemical machinery of the patient to elaborate by its own efforts the protective secretion which is required for the destruction of the invading bacteria." He employs a vaccine of a standardised strength, and preferably one obtained from the particular strain obtained from the patient. After an injection of this vaccine, there is a negative phase heralded by a crop of fresh pustules, but the phagocytic power, in a successful case, is so increased as to lead to a cure.

It is difficult to explain the action that yeast taken internally has upon a suppurative acne, but the action in some cases is well marked, while in others it proves useless. It acts presumably by rendering the soil unsuitable for the bacilli.

